# Comparison study of silicon carbide coatings produced at different deposition conditions with use of high temperature nanoindentation

**DOI:** 10.1007/s10853-016-0476-5

**Published:** 2016-10-14

**Authors:** Nadia Rohbeck, Dimitrios Tsivoulas, Ian P. Shapiro, Ping Xiao, Steven Knol, Jean-Michel Escleine, Marc Perez, Bing Liu

**Affiliations:** 1 School of Materials, The University of Manchester, Oxford Road, Manchester, M13 9PL UK; 2 Clean Energy/Nuclear Services, Amec Foster Wheeler, 601 Faraday Street, Birchwood Park, Warrington, WA3 6GN UK; 3 Nuclear Research and Consultancy Group (NRG), PO Box 25, 1755 LE Petten, The Netherlands; 4 Commissariat a l’Energie Atomique (CEA), CEA/Cadarache, 13108 St. Paul lez Durance, France; 5 Institute of Nuclear and New Energy Technology (INET), Tsinghua University, Beijing, 100084 China

## Abstract

The elastic modulus and hardness of different silicon carbide (SiC) coatings in tristructural-isotropic (TRISO) fuel particles were measured by in situ high temperature nanoindentation up to 500 °C. Three samples fabricated by different research institutions were compared. Due to varied fabrication parameters the samples exhibited different grain sizes and one contained some visible porosity. However, irrespective of the microstructural features in each case the hardness was found to be very similar in the three coatings around 35 GPa at room temperature. Compared with the significantly coarser grained bulk CVD SiC, the drop in hardness with temperature was less pronounced for TRISO particles, suggesting that the presence of grain boundaries impeded plastic deformation. The elastic modulus differed for the three TRISO coatings with room temperature values ranging from 340 to 400 GPa. With increasing measurement temperature the elastic modulus showed a continuous decrease.

## Introduction

Silicon carbide (SiC) is an important technical ceramic that is widely applied due to its high hardness and temperature stability. Over the past years there have been extensive efforts to develop SiC for the nuclear environment. Thus in the future, SiC-based composites could replace metal fuel cladding or fissile material is diluted within an inert SiC matrix to form a new type of fuel [1, 2]. The high temperature reactor (HTR) concept foresees the application of a fully ceramic fuel compact in which all fissionable material is completely encapsulated within composite tristructural-isotropic (TRISO) fuel particles consisting of successive layers of pyrolytic carbon (PyC) and SiC around the spherical kernel. In particular, the integrity of the SiC coating is crucial to ensure full retention of all radiotoxic compounds within the fuel in-service as well as during final disposal. Such SiC coatings are being produced by a fluidised bed chemical vapour deposition (FBCVD) process that can achieve dense, pure, and homogeneous coating layers. By optimising the coating parameters (temperature, precursor concentration), the desired microstructural characteristics can be obtained. Thus coatings vary in grain size and shape, texture, presence of residual porosity or amount of co-deposited second-phase free silicon or carbon. Even though our understanding of the relationship between deposition conditions and microstructural characteristics vastly improved over the past decades, some aspects in obtaining the best mechanical performance are still not clear. One crucial shortcoming is that the mechanical properties are usually assessed at ambient conditions, whereas a wide variety of applications use SiC in an elevated temperature environment. Using the high temperature nanoindentation technique it is possible to measure the mechanical properties of thin coatings in situ up to several hundred degrees celsius. Numerous publications have addressed the technical issues that arise when conducting reliable measurements at higher temperatures, but the data on SiC are still scarce [3, 4]. A short communication of our previous study reported the effect of neutron irradiation on a similar SiC specimen [5]. It was found that neutron irradiation at 1000 °C had caused some irradiation hardening, but no sizeable impact on the elastic modulus was measured. However, that study included only one SiC sample and thus no conclusions regarding the role of morphological features on the mechanical properties could be drawn. In addition, even though a few different research institutions have successfully produced TRISO particle fuel, few comparison studies have been published. The different sizes of the custom-built coating facilities and the variation in the fabrication parameters result in differing microstructural features, but the impact on the mechanical properties has not been identified yet. Here, we want to fill the aforementioned gaps by evaluating the influence of the microstructural characteristics on the elastic modulus and hardness up to 500 °C of three different SiC coatings fabricated by different research institutions. In addition to the high temperature nanoindentation tests, extensive microstructural characterization of the different SiC coatings, including scanning electron microscopy (SEM), electron backscatter diffraction (EBSD) and Raman spectroscopy, was undertaken to obtain an accurate description of the specimens and their respective microstructural characteristics.

## Experimental procedure

### Materials

The simulated TRISO particles measured in this study were fabricated by different research institutions (the manufacturer is indicated by the sample ID) and varied in their respective geometry and fabrication conditions (Table 1). In all cases ceramic surrogates were used as the initial substrate. Samples from the University of Manchester (UMAN, UK) and the Institute of Nuclear and New Energy Technology (INET, China) contained zirconia beads, whereas the particles from the Commissariat à l’énergie atomique (CEA, France) used alumina spheres as substrates. Initially the substrates were coated with a low and a high density pyrolytic carbon layer before fabricating the SiC layers. The CEA sample contained an additional high density carbon layer on the outside of the SiC coating, these particular specimens had been fabricated for the European PYCASSO experiments [6]. For means of comparison, nanoindentation experiments were also carried out on bulk SiC that was grown by static chemical vapour deposition (CVD), which was purchased from Rohm & Haas (Dow Chemical Company, USA).

**Table 1:** Samples for high temperature nanoindentation experiments: simulated TRISO fuel samples with SiC geometry and fabrication conditions

Sample	SiC thickness (µm)	FBCVD deposition conditions^a^	Microstructural characteristics	Comment
UMAN TRISO	35	1700 °C, MTS in hydrogen	Nano-sized grains along interface with the inner carbon coating, submicron grain size in the centre	Fabricated by UMAN
CEA TRISO	59	1550 °C, MTS in hydrogen	Nano-sized grains along the inner interface, elongated grains that can reach several microns in length in the centre of the coating	Fabricated by CEA [7, 8]
INET TRISO	31	1560 °C	Uniform submicron grain size throughout the full coating, some porosity	Fabricated by INET
Bulk CVD SiC	2 mm disc	Static CVD	Large, randomly oriented grains with sizes in the range of 5 to 50 µm	Thin disc cut from bulk piece CVD SiC, supplied by Rohm & Haas: *E* = 466 GPa and *ν* = 0.21 [9]

^a^Reference furnace temperature

### Microstructural characterization

Microscopic analysis was carried out with a Quanta 650 FEG microscope (FEI, USA) equipped with an EBSD Detector (NordlysMax from Oxford Instruments, UK). EBSD maps were obtained with a step size of 0.1 µm and the data were analysed using the Channel 5 software (Oxford Instruments, UK). SiC stoichiometry was evaluated by Raman spectroscopy using the Argon laser (514 nm) of a Renishaw 1000 Raman system with a 50 times objective. The elastic modulus evolution with temperature was measured by Raman spectroscopy [10, 11] similar to the description of Zhao et al. [12] by placing the sample onto a hot stage (Linkam Scientific Instruments, Tadworth, UK). For the Raman analysis of the indent imprints, the UV line (325 nm) of a Horiba LabRam System was used. The laser was focussed with a 40 times UV objective (NA = 0.5; Thorlabs, US). Each spectrum was taken in the range from 600 to 1100 cm^−1^ during 10 s and averaged over four individual accumulations. Raman spectra were fitted using the Lorentzian peak function in the Origin Pro 9.0 software (Origin Lab, USA).

### High temperature nanoindentation measurements

The high temperature nanoindentation experiments were carried out with a nanoindenter from Micro Materials Ltd., Wrexham, UK. Our earlier work described the facility and calibration procedure [5]. During tests at 300 °C or above a low and continuous argon flow through the chamber was established. The gas sensor (QRAE II, RAE systems, US) placed inside the chamber did not detect any oxygen, thus the residual oxygen level was below 0.1 vol%. Preliminary tests showed no change of the indenter tip shape up to 400 °C, but a difference was notable after testing at 500 °C, which was attributed to tip blunting due to oxidation. The area function had changed for low load indents on silica and hence a load of 100 mN in SiC was chosen so that minor changes at low indentation depth would not affect the extracted property values of hardness and elastic modulus. However, the observed tip degradation prevented any further increase in the measurement temperature. Here, the appropriate area function for the diamond tip of Berkovich shape was determined by the indirect method on fused silica before the beginning and after the end of the measurement of each sample [13].

**Figure 1:**
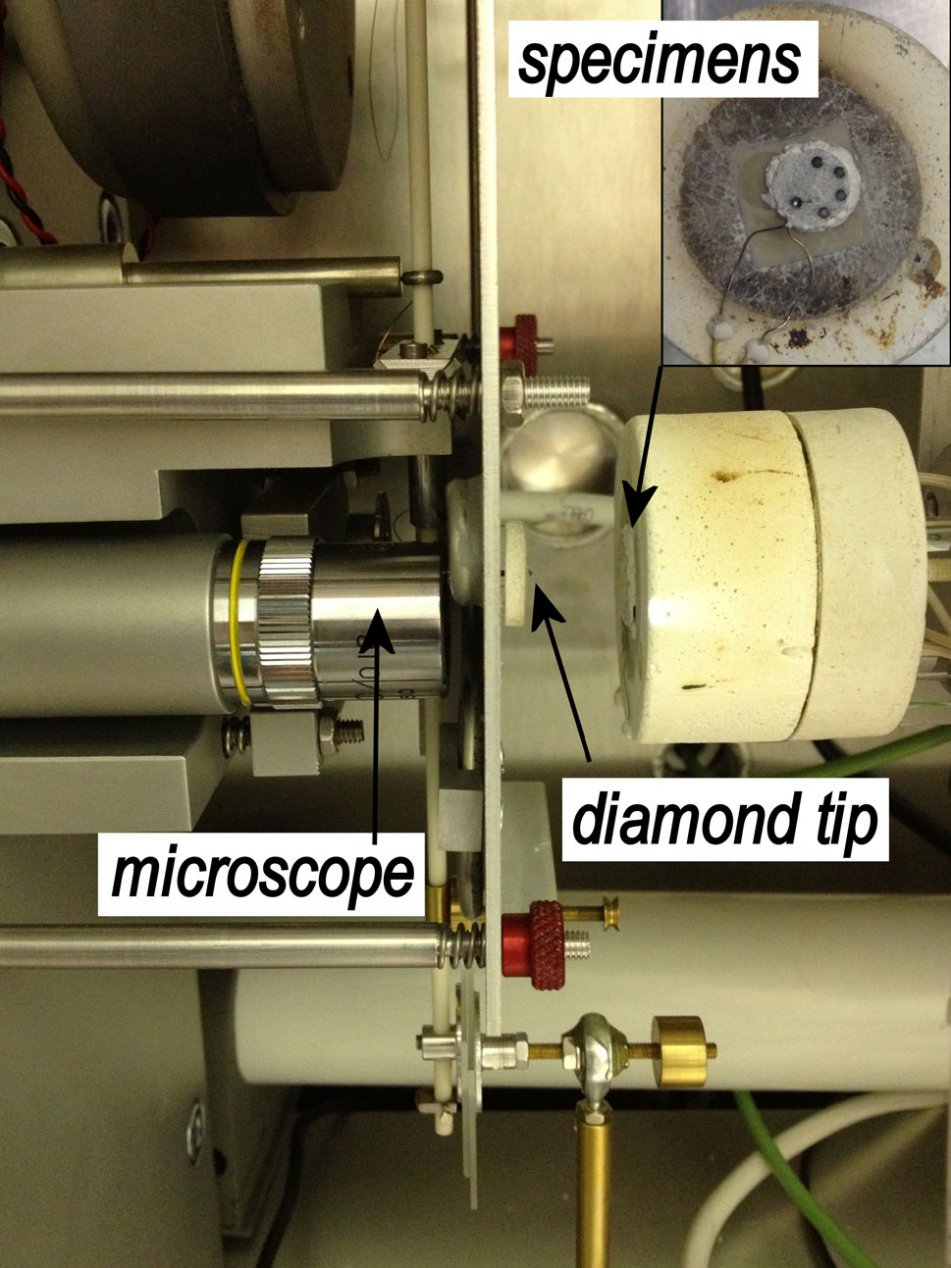
Photograph of the Micro Materials nanoindenter. The *inset* shows the TRISO particles cemented onto the hot stage

The TRISO particles were embedded, polished to the approximate cross section, and fixed onto the hot stage of the nanoindenter using high temperature cement. A photograph of the setup is shown in Fig. 1. Since the cement was water soluble all polishing was done without any water using firstly SiC grinding paper and finally with diamond paste (0.25 µm) on cloth. In addition to the SiC coatings, a thin disc of finely polished CVD SiC was used in the experiments. This piece was prepared by standard polishing procedures using colloidal silica suspension (0.05 µm) for the final surface finish.

During the nanoindentation experiments loading was performed over 20 s with a constant hold at maximum load of 10 s and a subsequent unloading over 10 s. The applied maximum load was 100 mN, which was found to produce indent impressions with diagonals >1.5 µm thus larger than the grain size of the SiC coatings. For the bulk CVD SiC, the experimental settings were the same although additionally tests with an increased maximum load of 500 mN were executed as well. At least 15 indents were taken at each temperature on different particles within the same sample. At higher measurement temperatures the instrument sometimes failed to detect the specimen surface correctly. Since those indents were easily distinguishable by an almost flat segment at the start of the load–displacement curve, they were excluded from the data analysis. For each single indent, thermal drift was measured during a 60 s holding period at the start of loading (pre) and after 90 % of the unloading cycle was completed (post). Only measurements with post-drift rates below 0.15 nm/s were considered acceptable and included in the analysis. The elastic modulus (E) and hardness (H) were calculated by applying the Oliver and Pharr method [14] using a temperature-adjusted elastic modulus value for diamond [15]. The Poisson’s ratio for diamond and SiC were 0.07 [15] and 0.21 [9], respectively.

Numerous issues complicate nanoindentation measurements at elevated temperatures and those have been addressed in detail in the literature [16–22]. Special care was taken to achieve temperature congruency of the specimen surface and the indenter tip. Therefore, an additional thermocouple was cemented onto the surface of the hot stage in close proximity to the specimen surface to monitor the specimen temperature more accurately. Upon contact between the tip and the sample surface a holding time of at least 60 s was programmed before each loading cycle, which reduced thermal drift rates to acceptable levels. Furthermore, changing temperatures can cause thermal transients in the electronics, which affect the depth signal measured by the transducer. Only extended holding times at constant temperatures can mitigate this effect, thus while the room remained at a constant temperature the tip and the specimen were heated to the measurement temperature at least 10 h before the first test was carried out. This procedure was found to be successful in achieving a similar spread of individual values within one set of nominally identical indents irrespective of the measurement temperature.

## Results

### Microstructural characterisation

**Figure 2:**
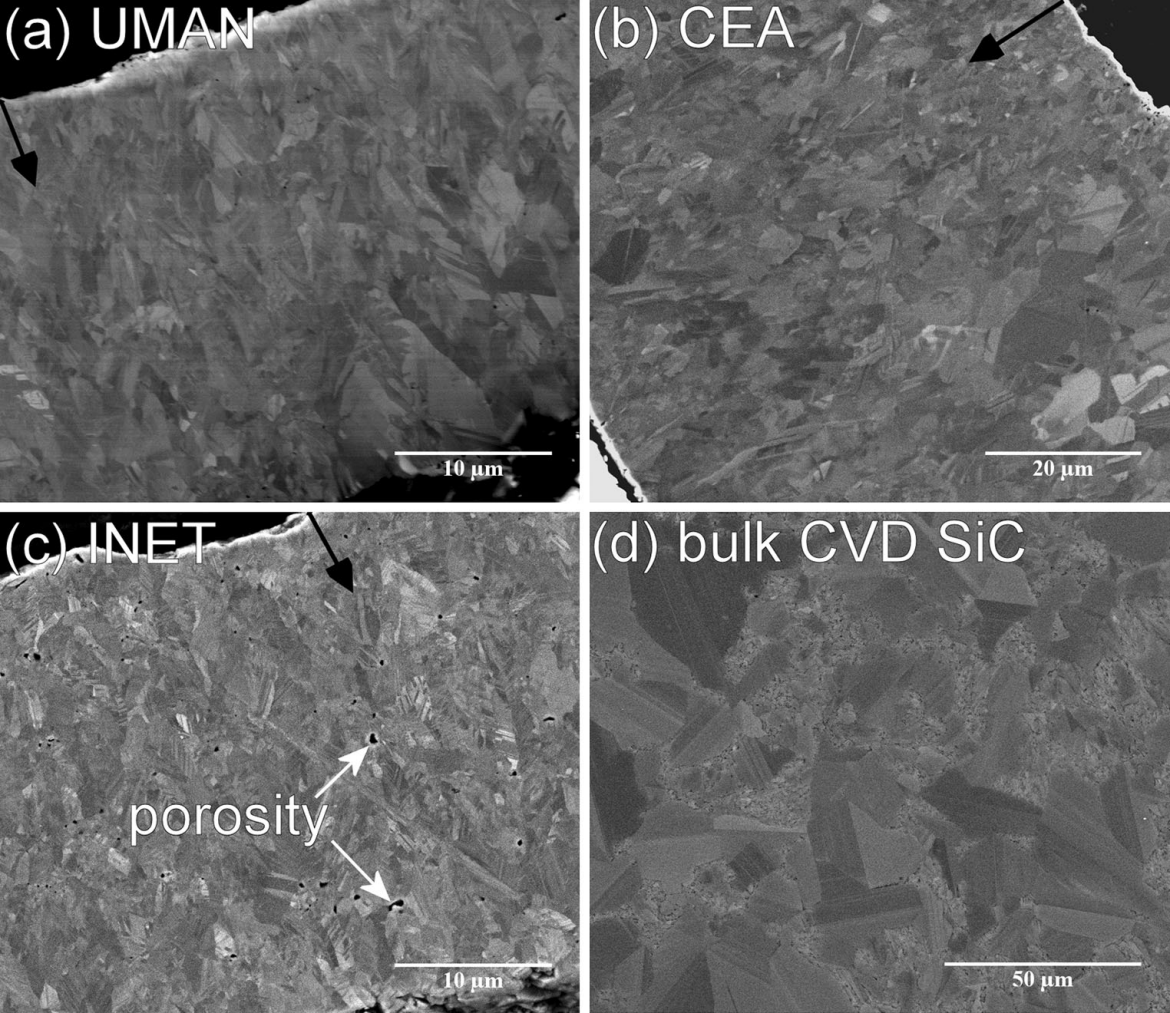
SEM micrographs taken on the polished cross section of the different SiC samples: **a** UMAN TRISO, **b** CEA TRISO, **c** INET TRISO, **d** bulk CVD SiC. The *black arrows* indicate the deposition direction of the SiC coatings

The temperature used during the CVD process strongly affects the microstructure of the final SiC coating, but the gas composition, precursor concentration, and furnace size also have an effect on the grain morphology, which in turn affects the mechanical performance. The three coating samples were fabricated using different FBCVD facilities and thus are expected to differ in their respective microstructure. SEM micrographs taken on the polished cross sections of the SiC coatings in the different TRISO particles are shown in Fig. 2. The typical grain morphology of TRISO coatings was found in the UMAN and CEA samples; both showed randomly oriented, nano-sized grains along the interface with the inner PyC layer, whereas grains grew in size and into a columnar shape along the deposition direction (Fig. 2a, b). The INET sample consisted of smaller grains that were more uniform in size throughout the complete coating with some visible porosity along the grain boundaries (Fig. 2c). The bulk SiC sample used as reference was fabricated by static CVD and it consisted of randomly oriented grains that were an order of magnitude larger than the SiC coatings with some grain diameters exceeding 50 µm (Fig. 2d).

**Figure 3:**
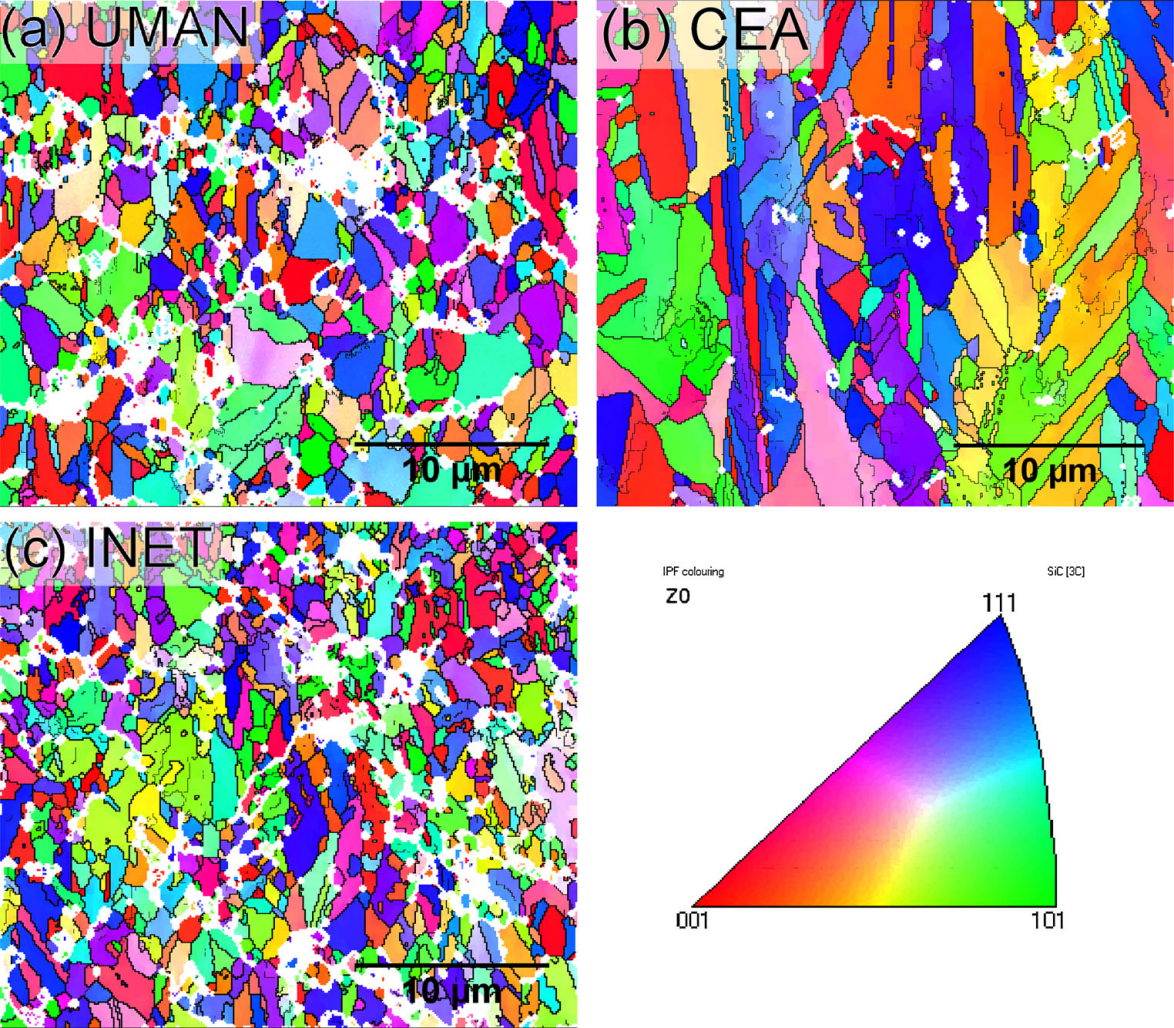
EBSD maps of TRISO coatings (deposition direction from *bottom* to *top*) **a** UMAN, **b** CEA, **c** INET. *Bold*, *black lines* represent high-angle grain boundaries, while thinner *grey lines* represent low-angle boundaries. Areas where indexing failed are shown in *white*

EBSD mapping offered more detailed microstructural information of the respective coatings and the difference in grain morphology was clearly evident. The maps shown in Fig. 3 were taken in the approximate centre of each coating’s cross section; this area was also used for the nanoindentation experiments. Compared with the UMAN and INET coating, the CEA sample consisted of significantly larger grains that were strongly elongated along the deposition direction. A statistical analysis of the data is given in Table 2. A minimum cut-off grain diameter was set to 0.3 µm in order to avoid the introduction of artefacts into the final results. Even though the grain morphology differed across all samples, analysis of the grain boundary (GB) character did not show any significant variations. As seen from Table 2, the fraction of grain boundaries with a high misorientation angle (>15°) was well above 60 % in all cases. On the other hand, low-angle grain boundaries (1.5° < *θ* < 15°) made up for only 4 or 9 % of the total. Additionally, all samples contained a high concentration of twin boundaries (Σ3), but the highest fraction was found in the larger-grained CEA coatings (see Table 2).

**Table 2:** Statistics extracted from EBSD maps

	Grain diameter (µm)	Fraction of Σ3 (%)	Fraction of GB misorientation (%)		
			High (>15°)	Low (1.5° < *θ* < 15°)	Non-defined (<1.5°)
UMAN	0.86 ± 0.57	29	71	4	25
CEA	1.38 ± 1.14	39	66	9	25
INET	0.80 ± 0.46	37	71	4	25

**Figure 4:**
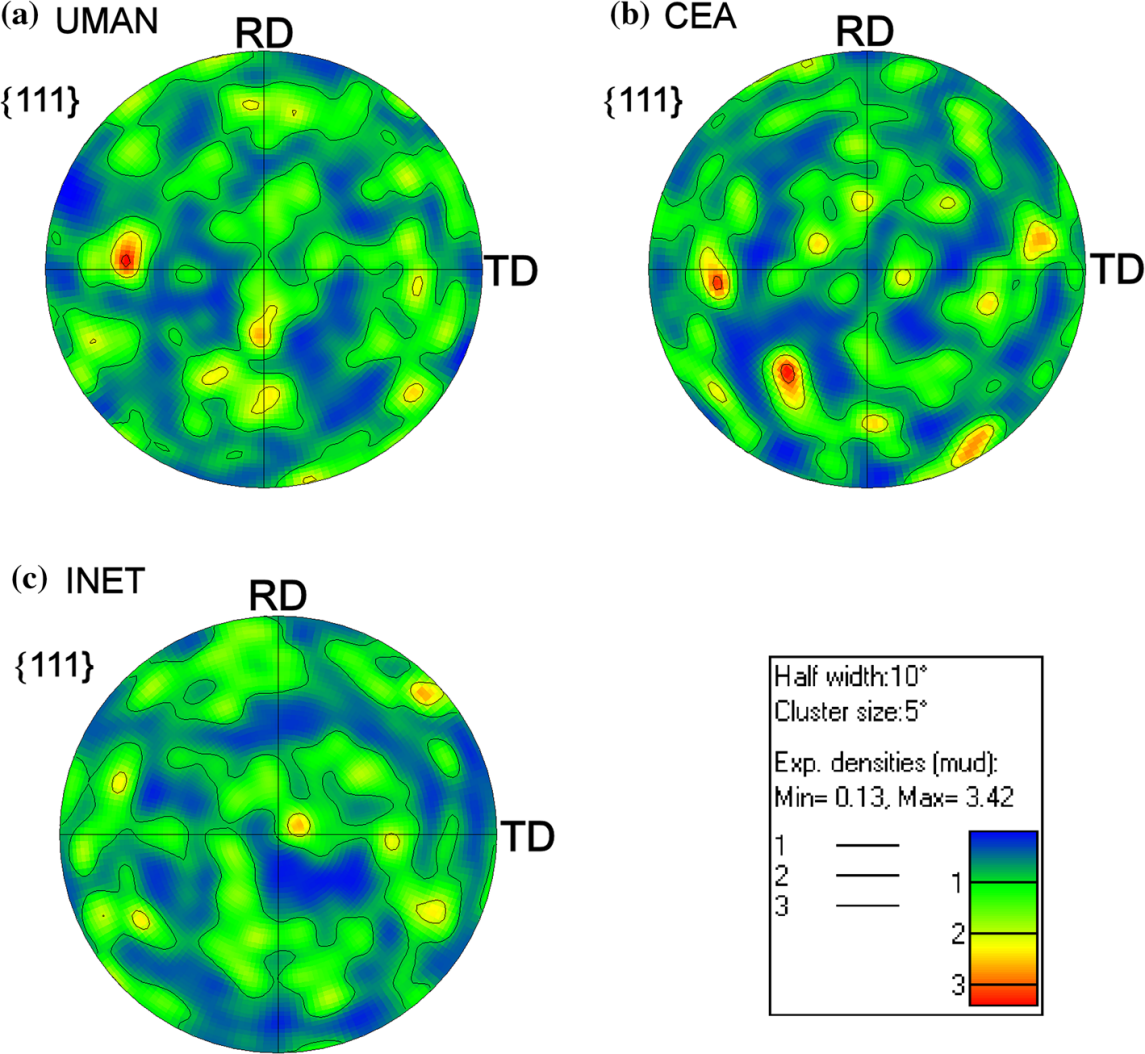
{111} pole figures generated from EBSD measurements on the three SiC coatings, showing weak texture in every case (the orientations RD and TD are rolling direction and transverse direction, respectively; RD is the grain growth direction in the EBSD maps of Fig. 3)

The EBSD maps did not exhibit an indication for a pronounced texture in any of the specimens, which was also found within other studies using EBSD analysis for TRISO fuel [23–25]. The {111} pole figures in Fig. 4 indeed do not show the prevalence of any preferred orientation. In all three pole figures, points of high intensity are small and randomly distributed indicating that they reflect spikes caused by a few large grains. Therefore, there is no evident tendency for the {111} planes to align with any of the main sample axes.

**Figure 5:**
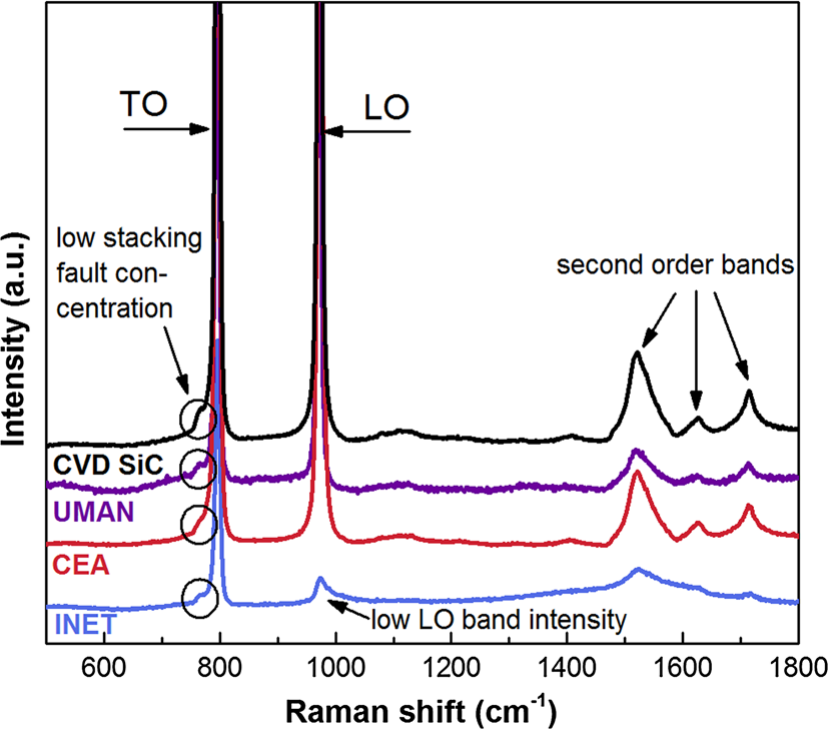
Raman spectra taken on the polished cross section of the SiC coatings and bulk SiC. Spectra magnified and offset for readability

Raman spectroscopy is a reliable and easily applicable method to evaluate the stoichiometry and crystallinity of the SiC coatings. Both characteristics are strongly dependent on the exact FBCVD fabrication parameters and Raman spectra were obtained from all samples measured here (Fig. 5). Overall, the spectra were found to be very similar and no traces of second-phase silicon or carbon were found. All samples were fully crystalline exhibiting the two sharp peaks for the transverse optical (TO) (796 cm^−1^) and zone-centre longitudinal (LO) (972 cm^−1^) bands of β-SiC. The second-order bands between ~1500 and 1750 cm^−1^ were also visible indicating a high crystallinity of the SiC. The stacking fault concentration was low in all samples as seen from the low intensity of the shoulder to the left of the TO peak. The only significant deviation was found in the INET coating, which showed a very low intensity of the LO band. Usually this is indicative for a strong preference of the (111) plane orientation, since the LO band is symmetry forbidden in that orientation. However, this was not confirmed by the EBSD analysis (pole figures in Fig. 4) and earlier Raman studies found that TRISO fuel fabricated by larger FBCVD systems often exhibited only a weak or no LO peak, but no correlation to the performance of these SiC samples was identified [26].

### Load–displacement curves of nanoindentation measurements

**Figure 6:**
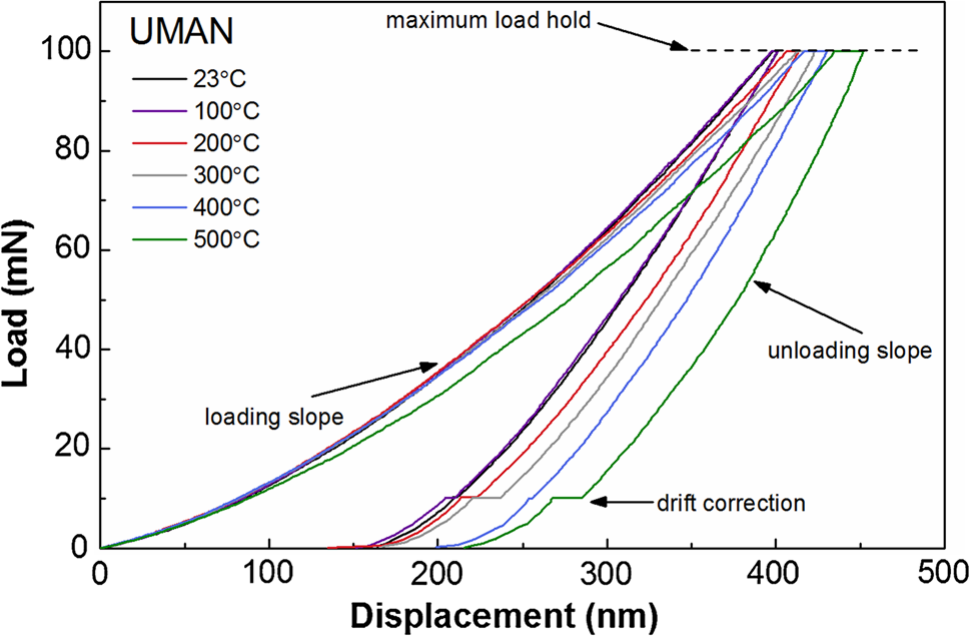
Selection of representative load–displacement curves from nanoindentation measurements of UMAN SiC coatings

Representative load–displacement curves taken at different temperatures on the UMAN SiC coating are shown in Fig. 6. For the chosen setting of the maximum load (100 mN) the maximum displacement was ~380 nm at ambient temperature. With increasing temperature the loading slope flattened and the holding period at maximum load led to an increased displacement over the same duration, which consistently moved the maximum displacement towards larger values at higher temperatures. This effect, hardly visible at 100 °C, was significant from 200 °C onwards. Though the measurement temperature did not affect the slope of the unloading curve visibly, some individual loading slopes showed sudden discontinuities that were likely related to displacement bursts, which in the past have been related to plastic deformation in SiC [27, 28]. But those were observed only in some measurements and could not be connected explicitly to specific occurrences such as cracking.

### Elastic modulus and hardness evolution with temperature

**Figure 7:**
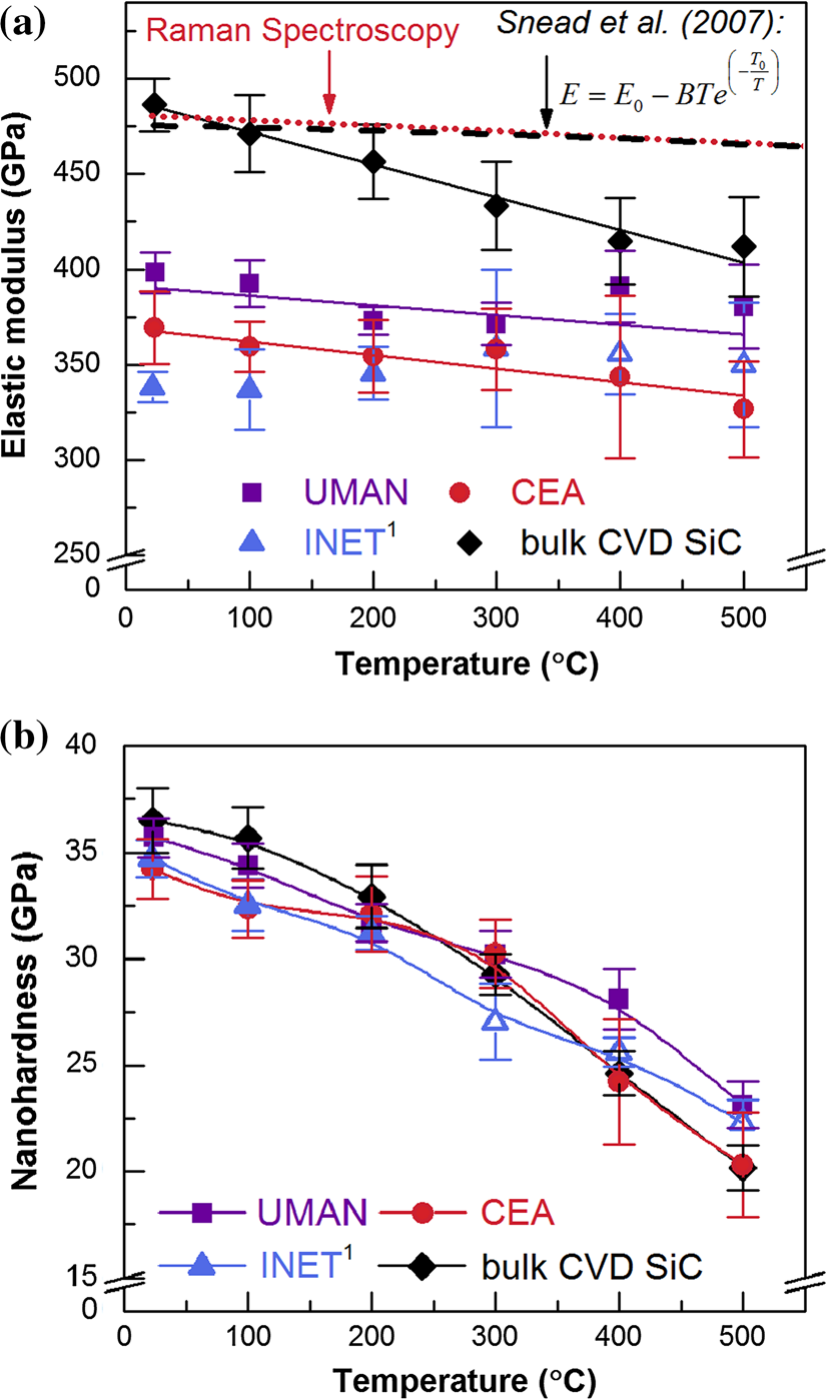
Results of the high temperature nanoindentation measurements: **a** Elastic modulus evolution with temperature. Nanoindentation data plots were linearly fitted; *red-dotted curve* from Raman spectroscopy measurements on the CEA sample, *black-dashed curve* from literature data according to Snead et al. [29]. **b** Hardness evolution with temperature.^1^ Due to higher drift rates the data points of the INET sample at 300, 400 and 500 °C are an average of fewer individual measurements (~10)

Figure 7 illustrates the results of the high temperature nanoindentation experiments. The elastic modulus evolution with temperature was measured additionally by Raman spectroscopy; those results were identical for all the TRISO samples here and hence only one curve (red-dotted line) is shown in Fig. 7a. Furthermore, a black-dashed curve was added, which was calculated from the empirical equation suggested by Snead et al. [29] in their review based on a range of macroscopical mechanical tests. The elastic modulus values of the UMAN and CEA coatings as determined by nanoindentation showed a slight decrease with increasing measurement temperature, which was approximated by a simple linear function [30, Eq. 1] with *T* as the measurement temperature and the fitted parameters *E*_0_ and *B*.

1 $$ E = E_{0} + BT $$

The temperature dependency was slightly more pronounced compared with the Raman data. After fitting the experimental results to Eq. [1], the Raman spectroscopy data gave a value of −0.027 for *B*, which was in agreement with earlier results [12]. The high temperature nanoindentation data yielded *B* = −0.05 ± 0.04 and −0.07 ± 0.04 for the UMAN and CEA samples, respectively. The INET sample did not exhibit the same behaviour, though these particular measurements showed slightly higher drift rates above 300 °C and hence the average has been taken from a lower number of individual measurements (~10). Thus this raises a concern about the statistic validity of that dataset. Using the same experimental settings during the nanoindentation tests as above, a room temperature elastic modulus value of 486 ± 16 GPa was determined for the bulk CVD SiC. This was slightly higher than the one quoted by the manufacturer (466 GPa [9]). The elastic modulus degradation with temperature was noticeably more pronounced in this bulk CVD SiC compared with the TRISO coatings leading to *B* = −0.17 ± 0.01.

All three different TRISO layers possessed a very similar hardness at room temperature (~35 GPa) and the degradation with temperature was similar in these cases (Fig. 7b). The CVD SiC exhibited a slightly higher value at room temperature (36.5 ± 1.5 GPa), but the decrease with increasing measurement temperature appeared to be more pronounced.

### Microstructural characterisation of the indent imprints

**Figure 8:**
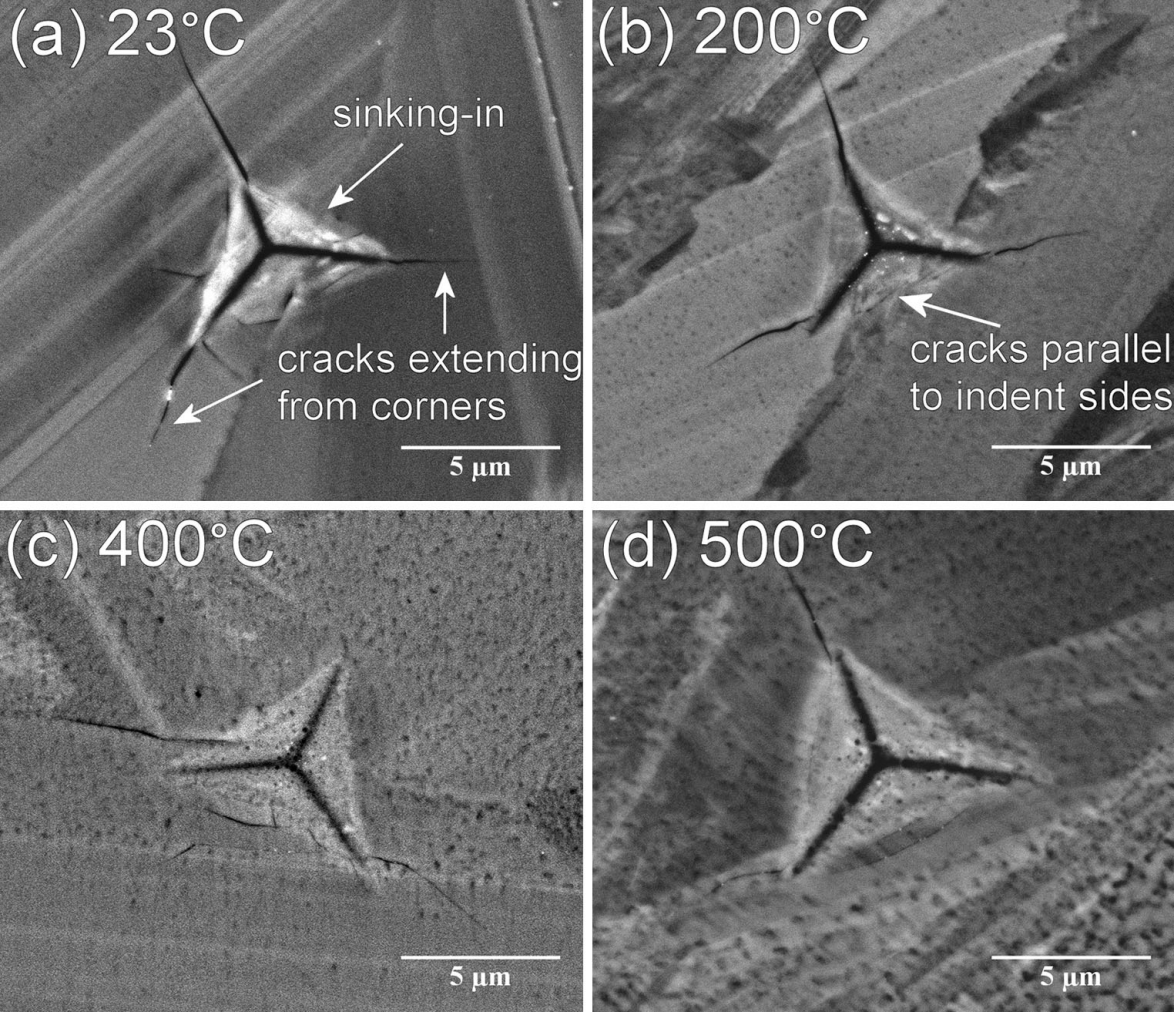
A representative selection of SEM micrographs illustrating indent impressions made at different temperatures with a maximum load of 500 mN in the bulk CVD SiC; **a** room temperature; **b** 200 °C, **c** 400 °C, and **d** 500 °C

Figure 8 gives a selection of SEM micrographs of the indent imprints made on the bulk CVD SiC with a maximum load of 500 mN. It was not feasible to capture good quality SEM images of the indent imprints in the SiC coatings due to the small feature size combined with the non-conducting cement embedding of the specimens.

The Berkovich diamond tip is an even, three-sided pyramid. As seen in Fig. 8a the indent sides had a slight concave shape indicating that some sinking-in had occurred [31]. This is often observed in ceramics where the plastically deformed zone is strongly contained around the indent and the elastic deformations are spread at a greater distance. The indent impressions showed cracks parallel to the indent sides extending from the three corners. The observed crack patterns were identical for indents made at room temperature and those up to 200 °C (Fig. 8a, b). Beyond that, however cracks formed more erratically and not necessarily extending from the three corners (Fig. 8c, d). The applied load for these specific indents was 500 mN, thus resulting in larger indent impressions, however, the cracking pattern as seen with the SEM was similar when a load of 100 mN was used.

**Figure 9:**
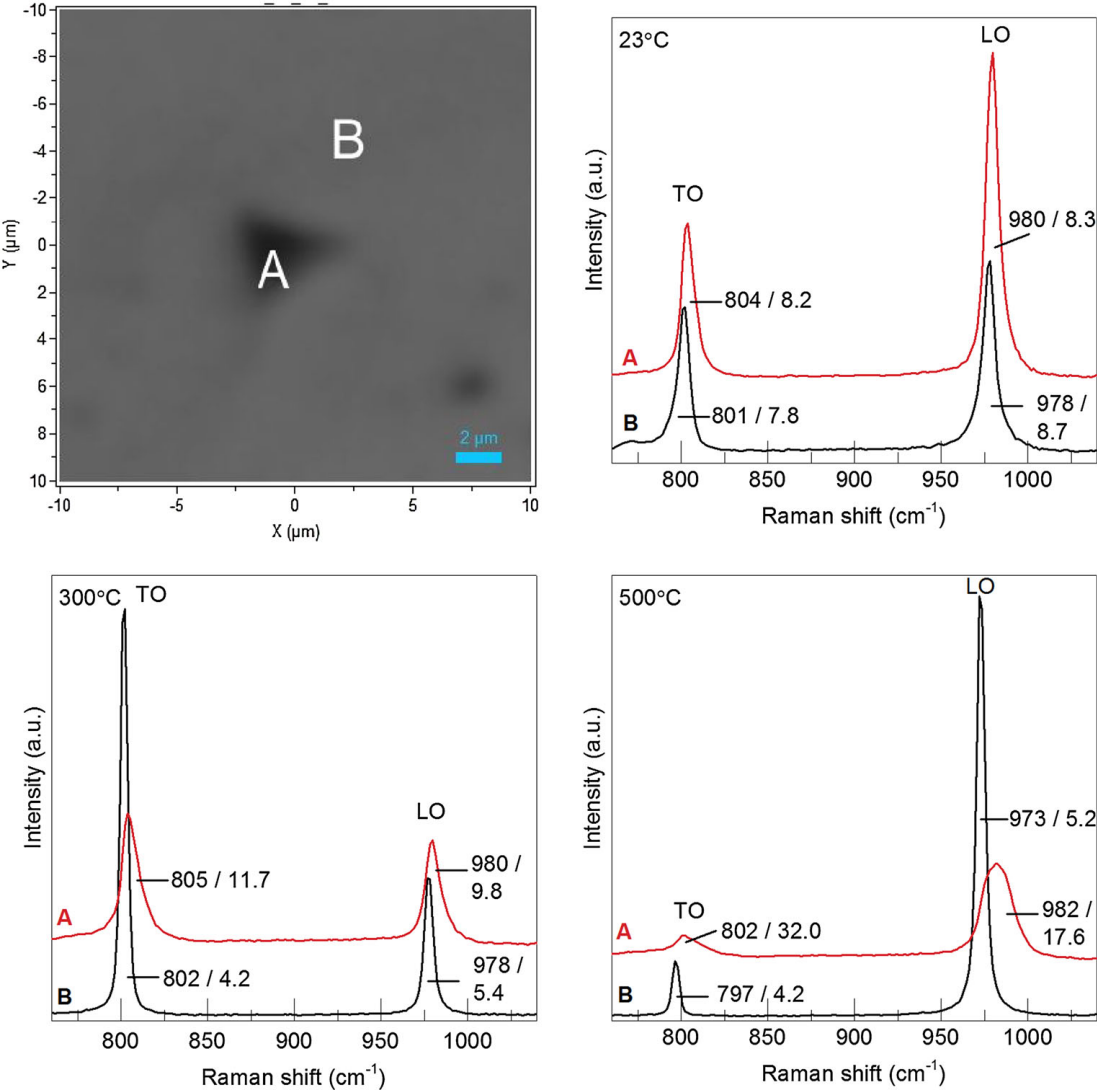
Raman spectra obtained from the indent imprints with the UV laser; **a** micrograph of an indent imprint illustrating the positions for the Raman measurement; **b**–**d** Spectra obtained from indent imprints at positions *A* and *B*, imprints were performed at 23 °C, 300 °C and 500 °C. All TO and LO bands are labelled with their respective positions and FWHM from Lorentzian fitting

Using Raman spectroscopy it is possible to evaluate the SiC morphology in a specific, small volume. Since the absorption coefficient of SiC increases exponentially with decreasing laser wavelength, a shorter laser wavelength greatly decreases the penetration depth into the material, which is more suitable when studying deformation close to the surface. The UV laser penetrates SiC by approximately 1.5 µm and with the objective used here the spot diameter is ~0.8 µm. In this Raman investigation the imprints of the 500 mN indents were measured, since the deformed volume was larger and thus providing more signal. Figure 9 shows two spectra for indents made at different temperatures. The spectrum shown in red was taken right in the centre of the indent imprint. At this point, the tip had penetrated furthest into the surface during the measurement and the most severe plastic deformation is expected to have occurred here. The second spectrum (shown in black) was taken at approximately 6 µm distance from this point as illustrated in Fig 9a. Irrespective of the measurement temperatures, the centre of the peaks shifted towards a higher wavenumber in the spectra taken in the indent centres. Such a shift is a sign for compressive stress in the material [32, 33]. For the indent made at room temperature the peak shift was clearly visible, but no change in the bandwidth or peak intensity occurred. For the indents made at higher temperatures both bands clearly widened. The full width at half maximum height (FWHM) in the 300 °C indent was twice that of the spectrum taken on the surface next to the indent. In the 500 °C indent, the TO band in the spectrum taken in the centre was only weakly visible and the FWHM of the LO band had tripled. Such a significant peak widening coupled with the strongly reduced intensity shows that the crystal structure is highly defective. None of the spectra showed any additional bands, thus there were no signs for either phase transformation or oxidation.

## Discussion

Nanoindentation is an established method for the evaluation of the mechanical properties of thin films and coatings and reports on SiC coatings at ambient temperature are readily available [34–36]. Measurements at elevated temperatures, however, pose technical difficulties (such as thermal drift of electronics, thermal mismatch, tip blunting) that have been well described in the literature [16, 19] and were mitigated cautiously during the experiments conducted here. Thus consistent values were obtained and the spread of individual results within one dataset of identical conditions was mostly similar to room temperature measurements. Great care was taken to identify a suitable sample preparation procedure for the spherical TRISO coatings and tests always included different particles within one sample for each temperature setting to eliminate effects caused by local variations in the specimen surface condition. This approach proved to be successful in obtaining comparable error bars for the coatings when compared with the bulk CVD SiC, suggesting that the spread of individual results was related to the inherent brittleness of SiC and not the sample preparation procedure. In our earlier work the technique of in situ nanoindentation at elevated temperatures was successfully applied for the evaluation of SiC coatings in TRISO fuel. Results showed the occurrence of irradiation hardening after SiC had been exposed to neutron irradiation [5]. Here, we use the same experimental approach to study how varying fabrication parameters that result in microstructures that differ in respective grain sizes and content of residual porosity affect the mechanical properties.

### Variation in grain size

Due to the varied fabrication parameters each SiC sample studied here exhibited a different microstructure. SEM and EBSD analyses identified the average grain diameter of the coatings in the range of 0.8 µm (INET) up to 1.38 µm (CEA), whereas the bulk CVD SiC, which had been fabricated by a static CVD process, possessed significantly larger grains with up to 50 µm in diameter. This variation, however, had little effect on the hardness value measured at room temperature, which was identical in the three TRISO coatings (~35 GPa) and only slightly higher in the bulk SiC piece (36.5 GPa).

**Figure 10:**
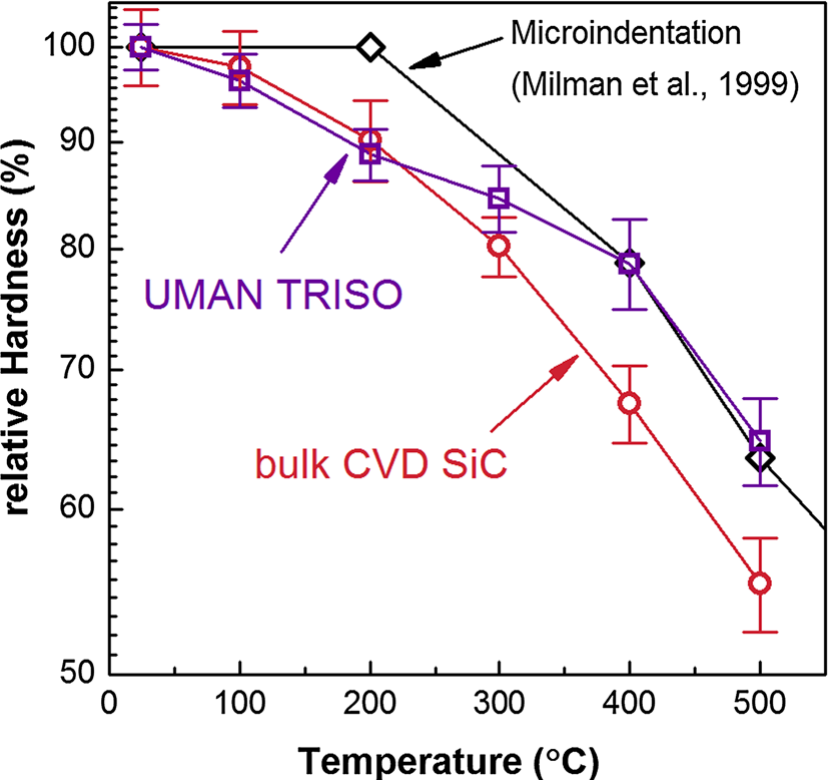
Hardness evolution with temperature, data normalised to room temperature hardness; literature data of fully dense, hot-pressed SiC taken from Milman et al. [3]

β-SiC exhibits a covalently bonded zinc blende structure in which dislocations must move across both ionic species to get back to an equivalent location in the crystal structure, thus the Burgers vector is large [37]. And since covalent bonds are directional, a great distortion of the bond angles is required during the propagation of a dislocation [38]. This is a difficult process to achieve and therefore the Peierls–Nabarro stress is high (7.5 GPa) [39] at low temperatures making SiC one of the hardest materials. However, with increasing temperatures thermal oscillation facilitates dislocation motion and the measured hardness decreases considerably. This was observed here as well since with increasing temperature the hardness dropped to a similar extent in all three TRISO coatings, although in comparison the drop in the bulk SiC was slightly more pronounced. This is shown in Fig. 10 where the hardness values were normalised to the initial room temperature value. There is no similar nanoindentation study available, so a comparison was made to a microindentation study (maximum load of 50 N) of dense, hot-pressed SiC by Milman et al. [3]. That former study determined a constant hardness up to 200 °C, but for temperatures above that the slope showed a similar decrease to the data obtained here. The authors concluded that within the temperature region below 200 °C, the hardness values were determined by the fracture stress of SiC and thus remained constant and only above this threshold the measured hardness was related to the yielding of SiC. Other studies measuring the hardness with temperature of α-SiC found a higher threshold temperature of around 400 °C [40–42].

This explanation however is not accurate for the experiments undertaken here. Earlier studies have shown that even at room temperature, the initial plastic deformation in SiC during nanoindentation is related to dislocation activity [27]. With the small curvature radius of the indenter tip a strong hydrostatic stress field is induced in which the critical shear strength is exceeded before the tensile stresses reach the cleavage strength [28]. Considering that subsequent SEM imaging showed cracking around the indent imprints regardless of the measurement temperature, it could be concluded that for the temperature range investigated here nanoindentation measurements induced plastic deformation by dislocation activity as well as fracture. Using Raman spectroscopy it was possible to identify that the plasticity of SiC beneath the indenter increased significantly at higher measurement temperatures, which would account for the significant decrease in hardness. Furthermore, the observed drop in hardness was notably steeper in the bulk CVD SiC, which consisted of grains that were an order of magnitude larger than those in the different TRISO coatings. This could be an indication that the presence of grain boundaries impeded dislocation activity and in their absence plasticity had a more significant effect on hardness for bulk CVD SiC at higher temperatures.

### Impact of porosity

The deposition parameters during the FBCVD fabrication process were optimised to achieve stoichiometric and dense coatings, however, the formation of nano-sized pores residing along grain boundaries [43] cannot be fully avoided and thus SiC coatings in TRISO fuel often possess densities slightly below the theoretical density [44]. This effect was not quantified in the samples here, but SEM analysis revealed porosity in the micron range in the INET coatings, whereas none was visible in the other two coatings. The commercially obtained CVD SiC was fabricated by a static CVD process, which results in exceptionally pure and fully dense materials [9]. Nanoindentation measurements identified variations in the respective elastic modulus of each SiC sample. Thus the CVD SiC exhibited a notably higher value compared to the TRISO samples, and amongst these coatings the INET sample possessed the lowest elastic modulus. Porosity has a strong effect on the elastic modulus and is likely to account for the differences observed here. Considering that β-SiC exhibits a strong anisotropy in the elasticity of the various planes [30], variations in the preferred plane orientation due to fabrication conditions could affect the elastic modulus value of the overall coating as well. However, none of the coatings examined here showed a strong texture in the EBSD analysis, so this particular aspect could not be evaluated within this study.

In SiC the decreasing stiffness of the atomic bonds reduces the elastic modulus at elevated temperatures. Considering that the maximum test temperature here (500 °C) is well below the reported temperatures for phase transformations and thermal decomposition in SiC (>1600 °C), no sudden change, but instead a continuous decrease of the elastic modulus is expected to be seen in this temperature range. This could be confirmed by conducting measurements using Raman spectroscopy, which identified a small and steady decrease of the elastic modulus with increasing measurement temperature that was in excellent agreement with the literature data based on conventional macroscopic mechanical testing of CVD SiC [29] (see Fig. 7a). However, the peak position in the Raman spectrum is solely related to the vibration of the atomic bonds and thus not affected by local microstructural features such as porosity. This had a two-fold effect on the present results; firstly, higher values were extracted for the elastic modulus of the SiC coatings by Raman spectroscopy compared to nanoindentation, and secondly, the same elastic modulus values were measured for all three different SiC coatings, which suggested that microstructural differences were not accounted for. Whilst the scatter within the nanoindentation data introduced some uncertainty in the gradient, there was a significant trend for a reduction in the elastic modulus at elevated temperature in the UMAN, CEA, and bulk CVD samples. This trend was more pronounced than expected from the Raman and literature data, with the bulk CVD SiC exhibiting the steepest drop in the measured temperature range. The underlying cause for this discrepancy was not clear, but might be connected to the observed changes in the deformation behaviour during indentation as discussed above. In nanoindentation, the elastic response of a material is assessed within a small measurement volume that is subject to an extreme hydrostatic stress. Thus, this approach differs substantially from macroscopic bending testing techniques, since shear bands with associated dislocations and microcracks form to accommodate the plastic deformation [27, 28, 45]. With an increasing concentration of such defects in the volume beneath the indenter at higher temperatures the materials’ elastic response is likely to be affected. This could account for the differing temperature dependency observed within this nanoindentation study when compared with literature data using conventional mechanical bending tests. The authors are not aware of any other high temperature nanoindentation tests on SiC materials that would allow a comparison, but it is generally possible that the temperature dependency of the elastic modulus as determined by nanoindentation experiments is inherently different to that from other techniques.

## Summary and conclusions

In this study we have compared the elastic modulus and hardness up to 500 °C of three TRISO coating layers and one bulk CVD SiC. The parameters during the fabrication of these coatings differed and thus the respective grain size was found to vary as well. Only one of the samples exhibited micron-size porosity visible in the SEM analysis and this sample also had the lowest elastic modulus (340 GPa) of the three coatings, whereas the fully dense bulk SiC exhibited the highest value with 480 GPa. The elastic modulus obtained by nanoindentation showed a stronger reduction with increasing temperature when compared to other testing techniques, which was attributed to increasing plasticity in the SiC occurring during indentation at higher temperatures. Irrespective of the grain size the hardness was found to be similar for all three coatings (35 GPa) and slightly higher in the bulk SiC (36.5 GPa). It decreased significantly with temperature and the drop appeared to be more pronounced in the larger grained bulk SiC suggesting that the grain boundaries impeded deformation. A subsequent analysis of the indent imprints using Raman spectroscopy showed a severely deformed structure, when the SiC was indented at higher measurement temperatures, thus indicating plastic flow even at these only moderately high temperatures.
